# Ultrasound-Guided Pulsed Radiofrequency Treatment for Meralgia Paresthetica

**DOI:** 10.7759/cureus.22015

**Published:** 2022-02-08

**Authors:** Margarida Costa Pereira, José Luís Carvalho

**Affiliations:** 1 Physical Medicine and Rehabilitation, Centro de Reabilitação do Norte, Vila Nova de Gaia, PRT; 2 Intervention and Musculoskeletal Rehabilitation Unit, Centro de Reabilitação do Norte, Vila Nova de Gaia, PRT

**Keywords:** ultrasound-guided injection, neuromodulation, mononeuropathy, lateral femoral cutaneous nerve, pulsed radiofrequency, meralgia paresthetica

## Abstract

Meralgia paresthetica (MP) is one of the most common mononeuropathies of the lower limb, characterized by injury or compression of the lateral cutaneous femoral nerve at the level of the anterior superior iliac spine and inguinal ligament. Many predisposing factors, such as weight gain, obesity, and restrictive clothing, contribute to the injury of the lateral cutaneous femoral nerve along its course from the pelvis towards the thigh. Although a great number of cases are successfully treated with conservative measures, a subgroup of patients suffer chronic dysesthetic pain with intermittent flare-ups in their lifetime, with a negative impact on quality of life, requiring additional treatment. The purpose of this case report is to describe the successful management of MP with ultrasound-guided pulsed radiofrequency of the lateral cutaneous femoral nerve.

## Introduction

Meralgia paresthetica (MP) is a painful condition characterized by injury or compression of the lateral femoral cutaneous nerve (LFCN) at the level of the inguinal ligament and anterior superior iliac spine (ASIS). [1] LFCN is a purely sensory nerve, arising from L1-L3 nerve roots. It courses laterally along the psoas muscle, crossing the iliac muscle and toward and medial to the ASIS. It courses laterally along the psoas muscle, toward the anterior aspect of the iliacus muscle; usually, exiting the pelvis just posterior to the inguinal ligament and medial to the ASIS. [2] This entrapment is usually related to a series of factors such as trauma, pelvic tumors, prolonged leg/trunk hyperextension or standing, pregnancy, external compression by belts or tight clothing, and weight gain. It can also be iatrogenic, as a complication from some surgical procedures. [3, 4] Sensory polyneuropathies, such as diabetes, can also involve the LFCN. Clinically, it is characterized by pain, paresthesia, dysesthesia, and numbness in the anterolateral thigh, as a direct result of LFCN compression. [1] Incidence is 4.3 per 10.000 persons-years [5]. Diagnosis is primarily clinical, based on history and physical exam findings. However, in less clear or atypical cases, diagnostic studies can be useful, such as ultrasound, magnetic resonance imaging (MRI), computed tomography (CT) scan, and electrophysiological testing, in particular, to rule out other conditions that may mimic MP - such as tumors and lumbar radiculopathy. Electrophysiological studies may be useful, as intact motor innervation rules out root disease. [6] A positive LCFN block - fast and temporary relief of symptoms - has been described as a specific diagnostic test for MP. Conservative treatment is effective in the vast majority of cases - in a series of 277 patients, it was successful in 91%. [7] Conservative treatment entails removal of the cause of the impingement - weight loss, avoidance of belts, and tight-fitting clothes. Rehabilitation may also facilitate healing of the LCFN, with physical agents and soft tissue mobilization. An LFCN block with local anesthetics and corticosteroids may also be tried, and adds the benefit of providing diagnostic insight; however, its effect is temporary. In case of conservative management failure, surgical options are neurolysis or neurectomy [8]. Therefore, a gap exists between conservative measures and surgical treatment, with a lack of options to offer to refractory cases. Search for alternatives in refractory cases led to a growing interest in neuromodulation to the LFCN using pulsed radiofrequency (PRF).

## Case presentation

We present the case of a 67-year-old male, with a history of glaucoma, dyslipidemia, and benign prostate hypertrophy. He was sent to our clinic because of complaints of paresthesia in his left thigh, worse with sitting or prolonged standing, with five years duration. He had no back pain and no strength deficits in the lower limbs. In the physical exam, an area of hypoesthesia circumscribed to the anterolateral left thigh was present, with no other abnormality in the neurological exam. No treatments of any nature had been tried before. Electromyography was performed, which revealed no signs of denervation of the L3 to S1 myotomes. He underwent a lumbar spine MRI, with no significant findings. A presumptive and clinical diagnosis of meralgia paresthetica was made. A left LFCN block was performed, under ultrasound guidance, using 1 cc of methylprednisolone 40 mg/ml plus 4 cc of ropivacaine 0.2%, which was effective in providing complete relief. The patient was seen in the clinic two months after and reported significant improvement in his pain. At eight months follow-up, a relapse had occurred, however, with lower intensity and frequency than reported at presentation. At this time, pulsed radiofrequency neuromodulation of the LFCN was proposed and explained, and the patient gave consent.

The patient was placed in the supine position; the area above the ASIS was identified and the skin above and below was prepped with betadine. A 3.7-13 MHz linear ultrasound probe (from a Logiq® P8 ultrasound machine), sterilized, and the sterile gel was used. The transducer was first placed at 2 cm inferior and medial to the ASIS, and the LCFN was identified as a small hypoechoic fascicular structure, between the tensor fasciae latae muscle and the sartorius muscle, 1cm deep to the skin surface. Using an in-plane technique, a disposable 22-gauge, 10 cm radiofrequency cannula (Boston Scientific/Cosman®) with a 10 mm active tip was inserted aiming at the LCFN.

The introducer needle was then withdrawn and the radiofrequency electrode was advanced. Sensory stimulation was performed using a Cosman G4 radiofrequency generator and provided confirmation of the correct radiofrequency electrode placement. Two cycles of pulsed radiofrequency were then undertaken, with temperatures of 42ºC for three minutes each. Finally, 1 mL of 0.2% ropivacaine and methylprednisolone 40 mg were injected through the radiofrequency cannula. The patient reported no significant discomfort or pain during the procedure or afterward. Six weeks later, he was asymptomatic, rating 0 out of four in the Douleur Neuropathique (DN4) questionnaire. He relapsed at eight months (two out of four in the DN4 questionnaire), at which time another treatment with radiofrequency was performed, in the same lines as described above. No side effects were reported in either radiofrequency treatment. The patient remained satisfied with the treatment and the results.

## Discussion

MP is one of the most common mononeuropathies of the lower limb. Many predisposing factors, such as overweight and belt use, contribute to injuring the LCFN along its course from the pelvis towards the thigh. As LCFN is a purely sensory nerve, its injury leads to purely sensory symptoms - pain, numbness, and paresthesia or dysesthesia - in the anterolateral aspect of the thigh. Although a great number of cases are successfully treated with conservative measures, a subgroup of patients suffer chronic pain with intermittent flare-ups in their lifetime, with a negative impact on quality of life. [8,9]

Neuromodulation via PRF is gaining popularity in the management of chronic pain. In particular, reports of prolonged analgesia provided by PRF in cases of painful mononeuropathies are increasing in the literature. PRF applies a brief, low-energy electrical stimulation with a long resting phase; in contrast to continuous radiofrequency (RF), PRF is not ablative, but instead neuromodulating, by a magnetic field generated by the electric stimulation. [10] The mechanism of PRF is still under study; however, there is evidence that PFR produces selectively larger lesions in the smaller principal sensory nociceptors, such as the C and Aδ fibers than in the larger non-pain related sensory fibers, such as Aδ fibers. [10] In addition, PFR modulates various pathways involved in nociceptive signaling, immune activity, and synaptic function. [11]

For the success of this technique, correct needle placement is crucial. As the location of the LCFN is variable [12], the landmark approach requires multiple needle passes. Ultrasound allows a more accurate and fast needle placement, with less discomfort for the patient. The use of ultrasound in the field of pain medicine has several advantages: direct visualization of soft tissue structures, allowing us to avoid potential complications by identifying blood vessels and viscera; constant visualization of the needle and its trajectory toward a specific target; and easy access in the clinic, with no need for a special set up.

## Conclusions

This case report illustrates the efficacy of PRF of the LCFN in the treatment of persistent MP, resulting in pain relief and high patient satisfaction, with no side effects. Moreover, it also demonstrates the advantage and accuracy of ultrasound as a guide to this technique. PRF is an effective and low risk treatment option in refractory MP. In the future, randomised and controlled trials regarding short and long term efficacy of PRF in MP are necessary.
